# Personal

**Published:** 1894-11

**Authors:** 


### Personal.

DR. J. C. SEXTON, of Rushville, Ind., has built and equipped a private hospital, which is provided with every convenience for the care of surgical and gynecological cases. The sanitary surroundings are all that could be desired. The heating is by natural gas. Each patient occupies a private room, and has a trained nurse in attendance. No contagious or venereal cases will be taken. Dr. Sexton's success in this line of practice and his high standing in the profession ought to insure the hospital a large patronage.

DR. I. S. STONE, of Washington, D. C., has removed to 1409 Rhode Island avenue, N. W., where he has opened a private sanitarium exclusively for the treatment of diseases of women The location is a desirable one, and the building is most complete in all its appointments.

DR. ALFRED E. DIEHL, of Buffalo, announces that he has opened an office at 362 Pearl street, where he will confine his practice to skin and venereal diseases, having devoted his attention to these specialties while abroad. Hours: 9–1 and 7–8.

DR. J. G. HARPER, of Buffalo, has removed from 190 Niagara street to the corner of Franklin and Huron streets. He announces diseases of women as a specialty. Hours: 9–10.30 A. M., 1–3 and 7.30 P. M.

